# Metabolic Changes in Focal Brain Ischemia in Rats Treated With Human Induced Pluripotent Stem Cell-Derived Neural Precursors Confirm the Beneficial Effect of Transplanted Cells

**DOI:** 10.3389/fneur.2019.01074

**Published:** 2019-10-22

**Authors:** Daniel Jirak, Natalia Ziolkowska, Karolina Turnovcova, Kristyna Karova, Eva Sykova, Pavla Jendelova, Nataliya Romanyuk

**Affiliations:** ^1^MR Unit, Department of Diagnostic and Interventional Radiology, Institute for Clinical and Experimental Medicine, Prague, Czechia; ^2^First Faculty of Medicine, Institute of Biophysics and Informatics, Charles University, Prague, Czechia; ^3^Faculty of Health Studies, Technical University of Liberec, Liberec, Czechia; ^4^Department of Neuroregeneration, Institute of Experimental Medicine, Czech Academy of Sciences, Prague, Czechia; ^5^Department of Neuroscience, Institute of Experimental Medicine, Czech Academy of Sciences, Prague, Czechia; ^6^Department of Neuroscience, Second Faculty of Medicine, Charles University, Prague, Czechia

**Keywords:** iPSC-NPs, stroke, magnetic resonance, metabolic changes, IMR, MRS

## Abstract

There is currently no treatment for restoring lost neurological function after stroke. A growing number of studies have highlighted the potential of stem cells. However, the mechanisms underlying their beneficial effect have yet to be explored in sufficient detail. In this study, we transplanted human induced pluripotent stem cell-derived neural precursors (iPSC-NPs) in rat temporary middle cerebral artery occlusion (MCAO) model. Using magnetic resonance imaging (MRI) and magnetic resonance spectroscopy (MRS) we monitored the effect of cells and assessed lesion volume and metabolite changes in the brain. We monitored concentration changes of myo-inositol (Ins), Taurine (Tau), Glycerophosphocholine+Phosphocholine (GPC+PCh), N-acetyl-aspartate+N-acetyl-aspartyl-glutamate (NAA+NAAG), Creatine+Phosphocreatine (Cr+PCr), and Glutamate+Glutamine (Glu+Gln) in the brains of control and iPSC-NP-transplanted rats. Based on initial lesion size, animals were divided into small lesion and big lesion groups. In the small lesion control group (SCL), lesion size after 4 months was three times smaller than initial measurements. In the small lesion iPSC-NP-treated group, lesion volume decreased after 1 month and then increased after 4 months. Although animals with small lesions significantly improved their motor skills after iPSC-NP transplantation, animals with big lesions showed no improvement. However, our MRI data demonstrate that in the big lesion iPSC-NP-treated (BTL) group, lesion size increased only up until 1 month after MCAO induction and then decreased. In contrast, in the big lesion control group, lesion size increased throughout the whole experiment. Significantly higher concentrations of Ins, Tau, GPC+PCh, NAA+NAAG, Cr+PCr, and Glu+Gln were found in in contralateral hemisphere in BTL animals 4 months after cell injection. Lesion volume decreased at this time point. Spectroscopic results of metabolite concentrations in lesion correlated with volumetric measurements of lesion, with the highest negative correlation observed for NAA+NAAG. Altogether, our results suggest that iPSC-NP transplantation decreases lesion volume and regulates metabolite concentrations within the normal range expected in healthy tissue. Further research into the ability of iPSC-NPs to differentiate into tissue-specific neurons and its effect on the long-term restoration of lesioned tissue is necessary.

## Introduction

Stroke is a neurodegenerative disorder and the leading cause of disability in adult humans. One in six people worldwide will have at least one stroke in their lifetime (1). There is currently no treatment for restoring lost neurological function after stroke. A growing number of studies have highlighted the potential of stem cell transplantation and more differentiated neural cell transplantation as intriguing therapeutic approaches to post-stroke reparation. Cells from different sources (fetal cortical- and striatal-derived neural precursors, subventricular zone precursors, neuroepithelial stem cells, bone marrow stromal cells, hESC-derived neural precursors, etc.) have been tested for their ability to reconstruct the forebrain and improve function after transplantation in animals with stroke (2–5). As an autologous, multifunctional cell therapy for stroke, induced pluripotent stem cell-derived neural precursors (iPSC-NPs) have significant potential. Several studies have demonstrated the beneficial effect of iPSC-NPs in ischemia treatment using animal models (6–9). It has been shown that transplantation of human iPSC-derived cells is a safe and efficient approach to promoting recovery after stroke, supplying the injured brain with new neurons for replacement (6). In one rat model study, pluripotent stem cells were shown to differentiate into more specific cortical neuronal progenitors, in turn differentiating into functional neurons, and improving neurological outcomes after intracortical implantation (10). Grafted human iPSCs-NPs have been shown to survive, differentiate into neurons and ameliorate functional deficits in stroke-injured neonatal (11), and aged brains (7). Their strong therapeutic potential has also been demonstrated in large animal models (9). However, the mechanisms of iPSC-NPs in stroke treatment have yet to be investigated in sufficient detail.

Magnetic resonance imaging and spectroscopy (MRI/MRS) are powerful non-invasive diagnostic tools used to provide anatomical and biochemical information. MRI is an optimal imaging modality for ischemic lesion visualization. MRS is a technique used in preclinical and clinical studies to obtain metabolic information of the brain parenchyma under experimental conditions. Although MRS cannot directly localize the area of transplanted cells, it can provide information on the metabolic processes of the brain following cell transplantation. The most frequently assessed metabolites in proton magnetic resonance spectroscopy (^1^H-MRS) are lactate, which accumulates as the end product of anaerobic glycolysis, and N-acetyl-aspartate (NAA), which is considered a predominantly neuronal marker (12, 13). Thus, reduction of NAA is often acknowledged as an indicator of neuron loss (14). However, alternative explanations, such as the metabolic degradation and redistribution of NAA, warrant further analysis (15).

In this study, we transplanted human induced pluripotent stem cell (IMR90)-derived neural precursors in a rat temporary middle cerebral artery occlusion (MCAO) model. As previously demonstrated in our laboratory, grafted cells successfully survive and provide trophic support to host brain tissue, leading to the recovery of sensorimotor, and motor functions (16). It should be noted, however, that these effects were mainly demonstrated in animals with small lesions, while animals with big lesions showed no improvement.

The aim of this study was to determine metabolic changes by ^1^H-MRS in the striatal tissue region of the rat brain within 4 months after focal brain ischemia. We studied the effect of transplanted iPSC-NPs on metabolite concentrations at measured time points (1 month and 4 months) by comparing changes in ischemic lesion size. The results presented here may help to elucidate the biochemical changes that occur during early stages of ischemic stroke, distinguish at-risk individuals, benefit early diagnosis, and improve our understanding of the dynamic pathogenesis of early cerebral ischemia (17).

## Materials and Methods

### Animals and Study Groups

All animal experiments were performed in accordance with EU Directive 2010/63/EU on the protection of animals used for scientific purposes, and approved by the ethics committees of the Institute of Experimental Medicine, the Czech Academy of Sciences, and the Institute for Clinical and Experimental Medicine.

**Figure 1:**
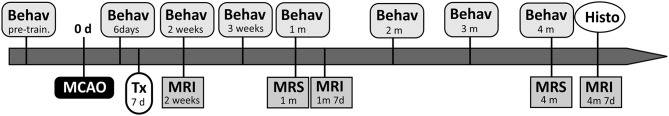
Schematic timeline of the experiments. The day when MCAO have been performed was taken as day 0. Animals were transplanted (Tx) with iPSC-NPs 7 days after lesion and were followed by magnetic resonance imaging (MRI), magnetic resonance spectroscopy (MRS), behavioral tests (Behav) over 4 months. Histological data (Histo) were acquired in the end of the experiment. D, days; m, months.

The study group consisted of 35 ten-week-old female Sprague-Dawley rats, including healthy controls (*n* = 3). Body weight ranged from 280 to 350 g to minimize differences in body size. All animals were pre-trained in the tape removal test for 3–4 days and tested for both behavioral tests the day before MCAO. Six days after MCAO, rats were randomly divided into control (*n* = 12) and transplanted groups (*n* = 20) and the last group began to receive the immunosuppression. Cells were transplanted 7 days after induction of the lesion. First MRI was performed 7 days after transplantation. According to its results, two existing groups were divided as follows: small control lesions without transplantation (SCL; *n* = 6), small lesions treated with iPSC-NPs (STL; *n* = 10), big control lesions without transplantation (BCL; *n* = 6), and big lesions treated with iPSC-NPs (BTL; *n* = 10). All these animals underwent MRI/MRS and behavioral tests according to the timeline shown in Figure 1, and immunohistochemical analysis was used at the end of the study of brain tissue. However, MRS data of several rats were excluded from the statistical analysis in accordance with the rules, which are described in the MRS section below.

### Human Induced Pluripotent Stem Cell-Derived Neural Precursors

The human iPSC line was derived from female fetal lung fibroblasts (IMR90 line, ATCC, USA) transduced with a lentivirus-mediated combination of OCT4, SOX2, NANOG, and LIN28 human cDNA [see (18)]. Clone selection, validation of the iPSC line and derivation of neuronal precursors are described in detail in Polentes et al. (16). Human induced pluripotent stem cell-derived neural precursors (iPSC-NPs) were routinely cultured in tissue culture flasks coated with poly-L-ornithine (0.002% in distilled water) and laminin (10 μg/ml in DMEM:F12), both obtained from Sigma (St. Louis, MO). Growth media comprising DMEM:F12 and neurobasal medium (1:1), B27 supplement (1:50), N2 supplement (1:100) (GIBCO, Life Technologies, Grand Island, NY), L-glutamine (2 mM) (Sigma), penicillin and streptomycin (50 U/ml) (GIBCO), FGF (10 ng/ml), EGF (10 ng/ml), and BDNF (20 ng/ml) (PeproTech, London, UK) were changed 3 times per week. Prior to implantation, the neural precursors were pre-differentiated in the same medium (omitting FGF and EGF) for 7 days.

### Antibodies and Immunocytochemistry

Pre-differentiated iPSC-NPs plated on poly-L-ornithine and laminin-coated chamber slides were washed in PBS and fixed with 4% paraformaldehyde in PBS for 15 min. Fixed cells were washed twice with PBS before staining. To identify neural precursors and differentiated neurons, antibodies directed against βIII-tubulin (1:100), Olig2 (1:400), nestin (1:200), chondroitin sulfate proteoglycan (NG2, 1:400), NF70 (1:200) (all Sigma-Aldrich), and Ki-67 (1:50) (Abcam) were used. To visualize primary antibodies, goat anti-mouse IgG conjugated with Alexa-Fluor 488 (1:400) and goat anti-rabbit IgG conjugated with Alexa-Fluor 594 (1:400) (Molecular Probes, Eugene, OR) were used. To visualize cell nuclei, DAPI was used. Confocal images were taken with the Zeiss LSM 5 Duo confocal microscope (Carl Zeiss AG). The index of the mitotic activity of the cells was evaluated as the ratio of Ki-67 positive cells to the total number of cells in the 10 randomly selected fields of view.

### Middle Cerebral Artery Occlusion Model and Cell Transplantation

Animals were anesthetised with 2.5% isoflurane (Forane^®^, Abbott Laboratories, Queenborough, UK). Transient focal cerebral ischemia was induced by intraluminal right middle cerebral artery occlusion (MCAO) using a nylon thread (0.08 mm diameter) and terminal cylinder of silicon (3 mm length, 0.24 mm diameter) for 90 min. Animals were then re-anesthetised for reperfusion and divided into control (*n* = 12) and transplanted (*n* = 20) groups. Based on volumetry results obtained by MRI, groups were further categorized according to lesion size.

Cell-treated animals were transplanted 7 days after ischemia induction. The control group was left untreated. Using an aseptic technique, a small hole was drilled into the skull above the lesion (0.5 mm anterior to the bregma, 3 mm lateral to the midline). Next, using a Hamilton syringe with its tip placed 4.5–5 mm deep from the cortical surface, 3 μl of a cell suspension (100 000 cells/μl) was slowly injected over a 5-min period into the lesion. Triple drug immunosuppression was used to prevent graft rejection. Sandimmun (10 mg/kg; intraperitoneal) (Novartis, East Hanover, NJ), Imuran (4 mg/kg; intraperitoneal) (Aspen Pharma Trading Ltd., Dublin, Ireland), and Solu-Medrol (2 mg/kg; intramuscular) (Pfizer Inc., New York, NY) were administered throughout the experiment (for 1 or 4 months). Immunosuppression administration started 1 day before cell transplantation and then was applied each second day (or three times per week).

### Behavioral Testing

The adhesive tape removal test (sensorimotor integration) was performed on iPSC-NP-transplanted animals and controls. A sticky colored paper dot (12 mm in diameter) was placed over the rat's left paw and fingers, and the time taken to remove the dot was measured. Rats were pre-trained for 3–4 days before MCAO and tested 3 times per week until the end of the experiment.

Apomorphine-induced rotation (motor dysfunction, pharmacological) was performed on all groups and quantified for 30 min after an intra-peritoneal injection of 1 mg/kg apomorphine in saline. Deficits were quantified using a formula modified from Arvidsson et al. (19): Score = IspiCyl+(2xIpsiHslf)-ContrCyl-(2xContrHslf) with IpsiCyl: rotation ipsilateral to the lesion around the central cylinder; IpsiHslf: rotation ipsilateral on itself; ContrCyl: rotation contralateral to the lesion around the central cylinder; ContrHslf: rotation contralateral on itself.

### Magnetic Resonance

MRI measurements were performed using a 4.7 T Bruker equipped with a custom-made surface coil. Rats were anesthetised by passive inhalation of 1.5–2.5% isoflurane in air. Breathing was monitored during the measurements. For imaging, a T_2_-weighted turbo spin-echo sequence (Rapid Acquisition with Relaxation Enhancement—RARE) was used. Sequence parameters were as follows: repetition time TR = 3,000 ms, effective echo time TE = 36.0 ms, turbo factor = 8, number of acquisitions AC = 8, field of view FOV = 3.5 cm, matrix 256 × 256, slice thickness 0.85 mm, number of slices = 29 (covering whole brain), voxel resolution 0.0159 μl, acquisition time 9 min 36 s. Imaging was performed 7 days, 1 and 4 months following iPSC-NP transplantation in treated groups. For the control groups the same time span was used for the unification, even if no transplantation procedure was carried out. Acquired images were used for anatomy reference and localization of spectra.

Following the imaging protocol, magnetic resonance spectroscopy was performed 1 and 4 months after MCAO. ^1^H-MR spectra were obtained from the striatal tissue of both hemispheres in all groups. They were then measured by the single-voxel PRESS MRS sequence with water suppression using the variable pulse power and optimized relaxation delays (VAPOR) technique according to the following parameters: TR = 2,500 ms, TE = 13.2 ms, AC = 512, acquisition time 21 min 30 s. The volume of interest was FOV = 4 ×4 ×4 mm^3^ (0.064 ml) for each hemisphere. Shimming for correction of magnetic in homogeneities was performed using an automated mapshim procedure. To get as robust data as possible, we had strict rules for data used in following imaging and spectroscopic analyses. Animal data were excluded if (1) there was movement artifacts due to breathing problems; (2) the half-width of water signal was 14 Hz or higher; (3) Cramér-Rao lower bounds (CRLB) was 25% and higher. To assess metabolic profiles of striatal tissue in both hemispheres, spectra were evaluated using the LC Model v 6.1 (20) to obtain metabolite concentrations. This software takes unsuppressed water as a reference. No corrections of calculated metabolite concentration for relaxation times were performed.

Concentrations of the following brain metabolites were used for analysis: Ins—a glial-specific marker (21) connected with cell metabolism and signaling pathways (22), it is also an osmolyte and essential requirement for cell growth (23); Tau—an osmoregulator and neuroprotectant with antioxidative properties that stabilizes the membrane, regulates cytoplasmic calcium levels, and reduces proinflammation (24, 25); GPC+PCh—are associated with membrane breakdown, myelinization/demyelinization or inflammation in brain diseases and were released in the brain with calcium overload (26); NAA+NAAG—markers of neuronal viability (27); Cr+PCr—PCr acts as a reservoir for the generation of ATP (23), this pair is also involved in antioxidative mechanisms and cellular bioenergetics (28); Glu+Gln—glutamate is the brain's major neurotransmitter and during ischemia acts as an important mediator of neuronal degeneration (29), also Glu+Gln pair decreased signals reflect glutamate-glutamine cycle dysfunction, while increased levels are connected with higher neurotoxicity and cell death in stroke (25, 30). Some metabolite values are presented together, as overlapping peaks from some of the spectra results made them difficult to distinguish.

### Volumetry and Experimental Groups

Images obtained by MRI were used for lesion volumetry analysis. Lesion volume was assessed by manually marking MR images using ImageJ software (version 1.46r, National Institutes of Health, USA) (31). According to initial lesion size measurements (14 days after MCAO and 7 days after iPSC-NP transplantation), animals were divided into two groups in both control and treated groups, with the dividing threshold at 0.027 ml (small lesion <0.027 ml < big lesion). Groups were divided into small control lesions without transplanted cells (SCL; *n* = 5), small lesions treated with transplanted cells (STL; *n* = 3), big control lesions without transplanted cells (BCL; *n* = 6), and big lesions treated with transplanted cells (BTL; *n* = 10). To make results more precise, lesion size was calculated as the mean value of the axial and coronal areas segmented in MR images.

### Histological Analysis

To analyse the fate of grafted iPSC-NPs, rats were sacrificed 4 months after transplantation. For the control groups the same time span was used for the unification, even if no transplantation procedure was carried out. The anesthetised animals were perfused with 4% paraformaldehyde in PBS (pH 7.4). Fixed brains were dissected and immersed in PBS with 30% sucrose. Frozen coronal sections (40 μm) were cut through the areas of interest. To identify human neural precursors transplanted into the rat brain, antibodies directed against human mitochondria (MTC02, 1:125) (Abcam) and against human nuclei (HuNu 1:40) (Chemicon, Temecula, CA) were used. For volumetric measurements, six sections were selected at 1-mm intervals along the craniocaudal axis, and whole images of the brain were taken with an Axioskop 2 plus microscope (Carl Zeiss AG, Oberkochen, Germany) and analyzed by ImageJ software (31). To follow the fate of the transplanted iPSC-NPs, antibodies directed against MAP2 (1:500), DARPP32 (1:500), Ki67 (1:50) (all Abcam), NeuN (1:100), TH (1:1,000) (Millipore, Billerica, MA), and GFAP (1:200) (Sigma-Aldrich) were used. To visualize primary antibody reactivity, the following appropriate secondary antibodies were used: goat anti-mouse IgG conjugated with Alexa-Fluor 488 (1:400) and goat anti-rabbit IgG conjugated with Alexa-Fluor 594 (1:400) (Molecular Probes, Eugene, OR). To visualize cell nuclei, DAPI was used. Confocal images were taken with the Zeiss LSM 5 Duo confocal microscope (Carl Zeiss AG). The index of the cell mitotic activity was calculated as the ratio of Ki-67-positive cells to the total number of surviving cells positive for HuNu. Cells were counted in the samples from 4 animals using at least 10 randomly selected fields of view for each animal. The total volume of the transplanted cell mass was estimated as described in Romanyuk et al. (32).

### Statistical Analysis

Statistical analysis was performed using GraphPad Prism 7 software. The following parameters were evaluated using non-parametric Wilcoxon matched paired test: metabolite concentration measurements of lesion and contralateral hemispheres at 1 and 4 months and comparisons of volumetry results at all-time points. To compare control and treated groups the non-parametric Kruskal-Wallis multiple comparison test with Dunn's multiple comparisons were used. Non-parametric Spearman correlation was estimated between lesion volume and metabolite concentrations from both time points and both hemispheres separately. For correlation and volume changes the Bonferroni correction was applied. Statistical analysis of RT-qPCR data was performed based on ΔΔCt values using GraphPad Prism software (GraphPad Prism 5, La Jolla, CA, USA). For all analyses *p* ≤ 0.05 was considered significant.

## Results

### Human Induced Pluripotent Stem Cell-Derived Neural Precursors Before and After Transplantation

**Figure 2:**
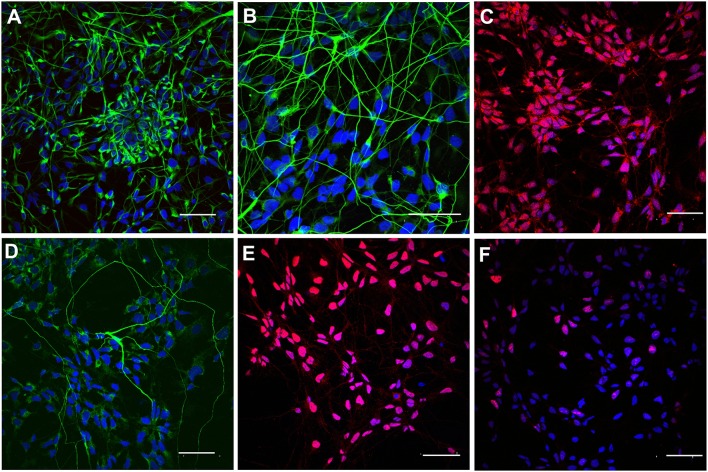
Immunocytochemical staining of pre-differentiated iPSC-NPs prior to transplantation. **(A)** Nestin, **(B)** βIII-tubulin, **(C)** NG2, **(D)** NF-68, **(E)** Olig2, **(F)** Ki67; magnification −20 μm.

In order to prepare iPSC-NPs for transplantation, cells were pre-differentiated over 7 days in a regular culture medium omitting FGF and EGF. According to immunocytochemical staining, the majority of pre-differentiated neural precursors were positive for nestin, βIII-tubulin, NG2, NF-68, and Olig2. Only 23.8 + 3.2% were positive for Ki67 (Figure 2). These data confirm their neural phenotypes prior to transplantation. For more detailed antigens and gene expression characteristics of pre-differentiated iPSC (IMR90)-NPs, see Romanyuk et al. (32).

**Figure 3:**
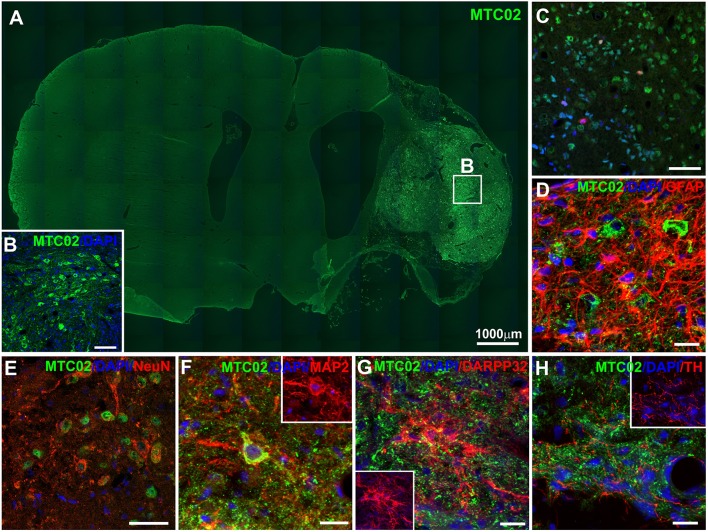
Immunohistochemical staining of ischemic rat brain tissue 4 months after iPSC-NP transplantation. Grafted neural precursors survived and migrated toward the lesion area **(A,B)**, differentiated into NeuN- **(E)**, MAP2- **(F)**, and DARPP32- **(G)** positive cells, actively integrated into the lesioned host tissue **(D,H)** and decreased their Ki67 index **(C)** to 3.2–0.8%.

Human iPSC-NPs in the ischemic rat brain were evaluated for survival and differentiation into neuronal and glial phenotypes 4 months after transplantation. The engrafted cells were identified by immunohistochemical staining for the human-specific markers HuNu and MTC02 (Figure 3). The severe necrotic processes occurring in big lesions complicated the quantitative estimation of the number of surviving grafted cells. Graphical information on transplant survival is presented in Supplementary Figure 1. Histological analysis of the survival, proliferation and differentiation of transplanted cells was performed in all transplanted animals where the graft survived (*n* = 12). Two of them are not presented in Supplementary Figure 1, because the graft survival was very poor. The total volume of the transplanted cell mass was 1.16 ± 0.12 mm^3^ for animals with big lesion and 0.70 ± 0.09 mm^3^ for animals with small lesion. Some grafted cells differentiated into more mature and tissue-specific neurons, such as NeuN-, MAP2- and DARPP32-positive cells (Figures 3E–G). A small portion of transplanted cells were positive for GFAP (Figure 3D), while transplants infiltrated by host cells were positive for GFAP or thyroxine hydroxylase (Figures 3G,H). Quantification of mitotic activity with Ki-67 immunoreactivity identified 2.53 ± 0.54% dividing cells, which was similar to the index shown previously for these cells (16, 32). Histological analysis of transplanted iPSC-NPs in animals with small lesions is presented on Supplementary Figure 2. No tumor formation was observed throughout the whole experiment. The survival of iPSC-NPs grafted in the rat brain was estimated from serial cross sections.

### Behavioral Testing

**Figure 4:**
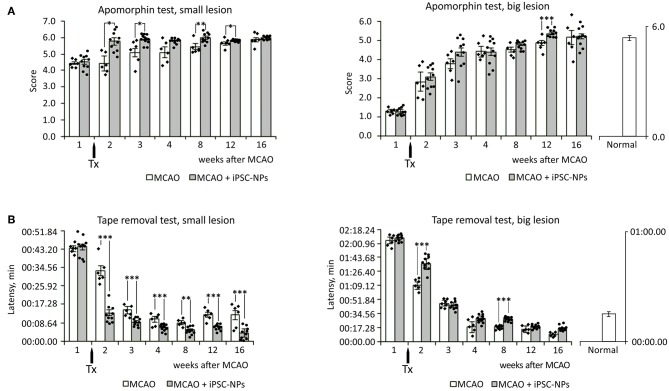
Transplantation of neural precursors improves functional recovery in animals with small lesion size. **(A)** Apomorphin-induced rotation test in non-grafted animals and animals grafted with iPSC-NPs. **(B)** Adhesive tape removal test in non-grafted animals and animals grafted with iPSC-NPs. Tx, transplantation. ^***^p < 0.001,^**^p < 0.01, ^*^p < 0.05.

To evaluate functional improvement after iPSC-NP transplantation in animals with big ischemic lesions, behavioral tests—the tape removal test and apomorphine test—were performed starting from the fifth day after lesions appeared (2 days before transplantation). Grafted animals showed almost no significant improvement compared to control animals with big lesions. Whereas, in animals with small lesions transplantation of iPSC-NP rescued functional impairment (Figure 4).

### Magnetic Resonance Imaging and Volumetry

Images obtained by MRI confirmed the effect of MCAO on brain tissue. Ischemic lesions were localized and assessed for both size and changes caused by iPSC transplantation at different time points. MR images were used in volumetric lesion measurements and for MRS localization.

**Figure 5:**
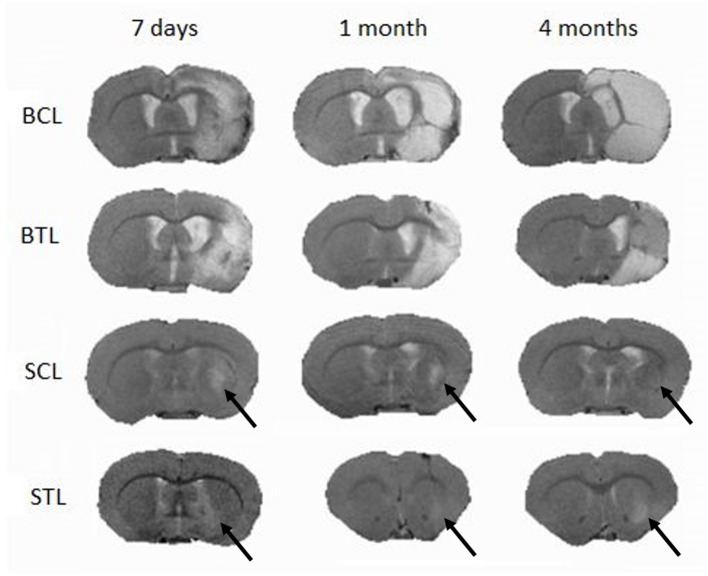
Typical MR images of lesions in the brain induced by MCAO following cell transplantation at three imaging time points (7 days, 1 month, and 4 months); SCL, small control lesions; BCL, big control lesions; STL, small treated lesions; BTL, big treated lesions. To present images representative for each time point lesion average size, not in all groups images are taken from one animal.

Focal brain ischemia volume measurements were performed at three time points: 7 days, 1 month, and 4 months after cells transplantation in the treated animals and at the same time points for the control groups. Lesion volume changes were observed at all-time points and in all groups. In the BCL group, lesion sizes increased and, especially between 1 and 4 months, signal became more homogenous. In the BTL group, lesion sizes increased at first (between 7 days and 1 month) but then decreased between 1 and 4 months. In the SCL group, lesions decreased throughout the experiment, reaching a value three times smaller than initial sizes after 4 months. In the STL group, lesion sizes decreased at first (between 7 days and 1 month) to a value three times smaller than initial sizes but then increased after 4 months. The volumetry results are summarized in Table 1. MR images representative for average results obtained from all time points in presented groups are contained in Figure 5.

**Table 1:** Volumetric measurement results.

**Group**	**Time point**	**Lesion volume [ml]**
SCL	7 days	0.012 ± 0.009
	1 month	0.006 ± 0.003
	4 months	0.004 ± 0.003
BCL	7 days	0.104 ± 0.06
	1 month	0.125 ± 0.07
	4 months	0.145 ± 0.09
STL	7 days	0.006 ± 0.009
	1 month	0.002 ± 0.0008
	4 months	0.011 ± 0.007
BTL	7 days	0.062 ± 0.06
	1 month	0.147 ± 0.02
	4 months	0.119 ± 0.06

*Data are expressed as the lesion mean [ml] ± std; SCL, small control lesions; BCL, big control lesions; STL, small treated lesions; BTL, big treated lesions*.

### Magnetic Resonance Spectroscopy

**Figure 6:**
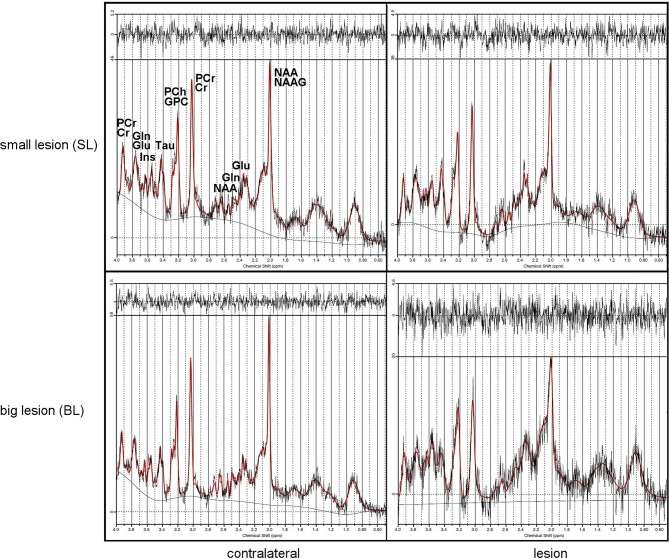
Typical MRS rat brain spectra showing lesions (right) and contralateral sides (left) from small (top) and big (bottom) lesions with signal intensities scaled to the highest signal in the spectrum.

^1^H-MR spectroscopy (^1^H-MRS) was employed to determine metabolic changes in the striatal tissue region of the rat brain over 4 months after focal brain ischemia. CRLB was usually around 10% in our study. Unfortunately, sometimes there was problem with shimming. It resulted in wide Full Width Half Maximum (FWHM) of the water signal. Linewidths of <0.1 ppm are considered essential for ^1^H-MRS *in vivo*; for 4.7T it is 20 Hz (33). We excluded data if FWHM > 14 Hz); usually FWHM was around 10 Hz in our study. The spectroscopy results are summarized in Tables 2A,B, with typical spectra given in Figure 6.

**Table 2:** MRS results of absolute metabolite concentrations in the striatal tissue: small lesions **(A)** and big lesions **(B)** groups.

**Small lesions**		**Ins**	**Tau**	**GPC+PCh**	**NAA+NAAG**	**Cr+PCr**	**Glu+Gln**
**(A)**							
1 month	C_C	5.5 ± 1.6^*^^§§§^	7.2 ± 1.8^**^^§§§^	1.8 ± 0.5^**^^§§§^	10.0 ± 2.2^**^^§§§^	8.8 ± 2.2^**^^§§§^	15.1 ± 4.1^**^^§§§^
	C_L	5.6 ± 2.1	5.0 ± 1.5^*^^§§§^	1.7 ± 0.6	7.8 ± 2.2^**^^§§§^	7.2 ± 2.5*^§§§^	12.7 ± 3.5^*^^§§§^^**^^§§§§^
	iPS_C	2.9 ± 0.8	5.0 ± 0.2	1.0 ± 0.0	6.0 ± 0.4	5.0 ± 0.2	9.2 ± 0.8
	iPS_L	4.1 ± 1.2	4.3 ± 0.7	1.2 ± 0.2	4.4 ± 0.6	4.7 ± 0.2	7.4 ± 0.4
4 months	C_C	5.3 ± 2.0	7.1 ± 2.5	1.4 ± 0.5	10.4 ± 4.4	8.2 ± 3.1	13.3 ± 5.5
	C_L	5.9 ± 2.3	4.2 ± 1.5	1.4 ± 0.5	7.8 ± 2.2	6.9 ± 2.3	10.1 ± 2.6
	iPS_C	3.4 ± 2.5	3.9 ± 3.1	1.0±−0.6	5.9 ± 4.2	5.1 ± 3.3	9.3 ± 6.5
	iPS_L	7.3 ± 7.0	5.1 ± 3.9	2.1 ± 1.3	4.9 ± 2.8	5.6 ± 2.6	10.0 ± 7.2
**Big lesions**		**Ins**	**Tau**	**GPC+PCh**	**NAA+NAAG**	**Cr+PCr**	**Glu+Gln**
**(B)**							
1 month	C_C	3.1 ± 0.6	4.6 ± 1.0	1.0 ± 0.2	5.9 ± 0.5	5.1 ± 0.4	8.2 ± 1.0
	C_L	2.7 ± 0.9	1.8 ± 0.5	0.6 ± 0.2	2.8 ± 1.2	2.1 ± 1.1	3.7 ± 1.8
	iPS_C	1.9 ± 0.9^*^^§§^	3.0 ± 1.2^*^^§§^	0.7 ± 0.2^*^^§§^	4.2 ± 1.6^*^^§§^	3.6 ± 1.2^*^^§§^	5.9 ± 1.6^*^^§§^
	iPS_L	2.0 ± 1.0	1.5 ± 1.0	0.5 ± 0.3	1.8 ± 1.2	1.7 ± 1.2	3.2 ± 1.6
4 months	C_C	3.3 ± 0.3	4.7 ± 0.6	0.8 ± 0.1	5.9 ± 0.7	5.2 ± 0.5	8.1 ± 1.1
	C_L	2.5 ± 1.0	1.6 ± 0.2	0.6 ± 0.2	2.7 ± 1.0	2.3 ± 0.8	4.0 ± 1.3
	iPS_C	4.7 ± 2.0	5.3 ± 1.3^**^^§^	1.3 ± 0.5	7.6 ± 2.6^**^^§^	6.5 ± 2.1^**^^§^	11.4 ± 4.4^**^^§^
	iPS_L	4.4 ± 4.2	2.9 ± 1.9	1.1 ± 0.8	2.8 ± 2.4	3.4 ± 2.8	5.6 ± 3.7

Data are expressed as the mean [IU] ± std. C_C: spectra from the contralateral hemisphere in animals with MCAO and without iPSC transplantation; C_L: spectra from lesions in animals with MCAO and without iPSC transplantation; iPS_C: spectra from the contralateral hemisphere in animals with MCAO and with iPSC transplantation; iPS_L: spectra from lesions in animals with MCAO and with iPSC transplantation; Wilcoxon matched paired test and Kruskal—Wallis tests were performed:

* *p <0.05*,

** *p <0.01*,

*** *p <0.001*,

§ *contralateral/lesion*,

§§ *1/4 months*,

§§§ *SCL vs. BTL*,

§§§§ *SCL vs. BCL; asterisks are informing about the tests significance while the rest of the signs are referring to the used comparison tests; asterisks denoting significance level indicate the first of the compared cells*.

**Figure 7:**
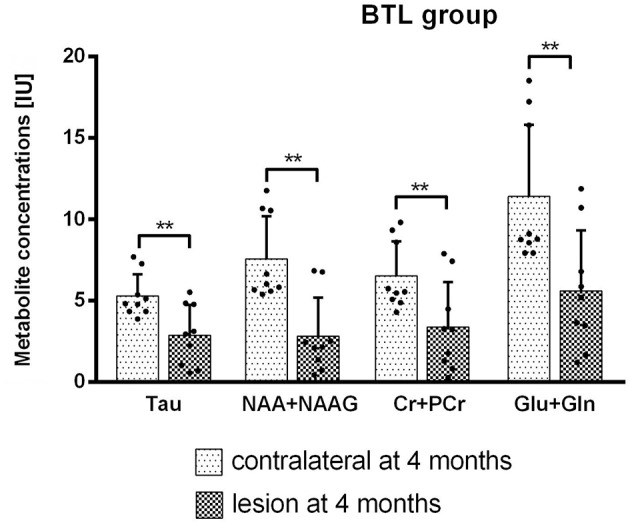
Metabolite concentrations from both hemispheres were compared using Wilcoxon matched paired test. BTL group at 4 months after MCAO. Significant results (*p* = 0.0039) were observed for Tau, NAA+NAAG, Cr+PCr, and Glu+Gln concentrations [IU].

MRS results from big lesion groups 1 and 4 months after MCAO revealed significant (*p* < 0.05) or at the limit of statistical significance (*p* = 0.0625) differences in metabolite concentrations [IU] between ipsi- and contralateral hemispheres. Significant differences were observed in MRS measurement 4 months post-MCAO in BTL group (Figure 7) in the following metabolites: Tau (*p* = 0.0039), NAA+NAAG (*p* = 0.0039), Cr+PCr (*p* = 0.0039), Glu+Gln (*p* = 0.0039). These changes are thought to occur due to the extent of ischemic injury. In the same group metabolite changes (Tau, GPC+PCh, NAA+NAAG, Cr+PCr, Glu+Gln) were observed to be on the limit of statistical significance (*p* = 0.0625) 1 month after occlusion was performed. Also in the BCL group results on the limit of statistical significance (*p* = 0.0625) were observed. Four out of six measured metabolite concentrations differed between ipsilesional and contralateral brain tissue in this group: Tau (4 months), NAA+NAAG (4 months), Cr+PCr (1 month and 4 months), and Glu+Gln (1 month and 4 months). In all four groups, metabolite concentrations were lower on the lesion side. The metabolite concentrations did not differ significantly between healthy animals and contralateral hemisphere in SCL group with MCAO and without iPSC transplantation. The absolute concentration of selected metabolites obtained from healthy animals were: Ins = 6.4 ± 0.9 IU, Tau = 8.0 ± 0.1 IU, GPC+PCh = 1.9 ± 0.3 IU, NAA+NAAG = 11.4 ± 2.4 IU, Cr+PCr = 9.8 ± 1.4 IU, Glu+Gln = 19.2 ± 4.9 IU (Supplementary Figure 3).

**Figure 8:**
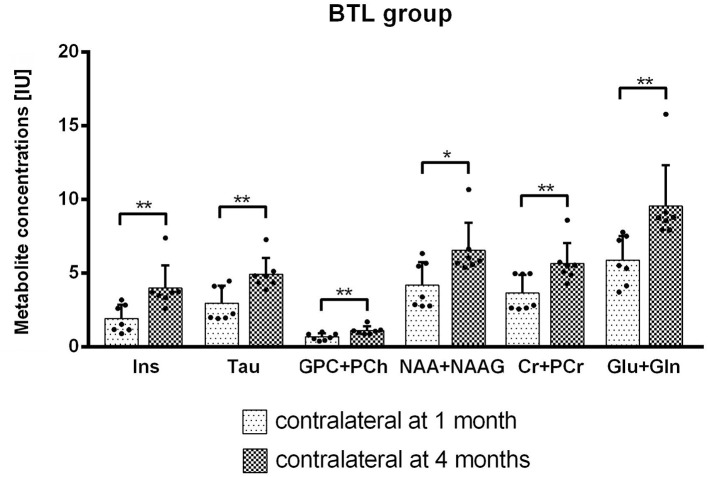
Metabolite concentrations [IU] for the contralateral hemisphere in time were compared using Wilcoxon matched paired test. BTL group from between 1 and 4 months after MCAO on the contralateral side. Significant results were observed for Ins (*p* = 0.0156), Tau (*p* = 0.0156), GPC+PCh (*p* = 0.0156), NAA+NAAG (*p* = 0.0313), Cr+PCr (*p* = 0.0156), Glu+Gln (*p* = 0.0156) concentrations [IU].

Metabolite concentrations changes between 1 and 4 months after MCAO were calculated using Wilcoxon matched paired test. In the BTL group significant results were observed on the contralateral side for Ins (*p* = 0.0156), Tau (*p* = 0.0156), GPC+PCh (*p* = 0.0156), NAA+NAAG (*p* = 0.0313), Cr+PCr (*p* = 0.0156), Glu+Gln (*p* = 0.0156) (Figure 8).

Spectroscopic results from all groups were compared, separately for both time points and both hemispheres using the Kruskal-Wallis multiple comparison tests with Dunn's multiple comparison test (Tables 2A,B). After 1 month, groups were observed to be significantly heterogeneous in results from all measured metabolites and in both hemispheres. The highest significant heterogeneity was obtained from NAA+NAAG (*p* < 0.0001) 1 month after MCAO on the lesion side. From this time point also between-groups significant differences were found. On the lesion side almost all metabolites (Tau, NAA+NAAG, Cr+PCr, Glu+Gln) concentrations were observed to differ between SCL vs. BTL groups using Dunn's multiple comparisons test. On the contralateral side this difference was found for all metabolites. Four months after lesion, results from ipsilateral hemisphere of MCAO groups were significantly inhomogeneous in NAA+NAAG and Cr+PCr, while no inhomogeneity was found on the contralateral hemisphere at this time point. There were no significant differences found between the control and treated groups with the same lesion sizes. Although the mean metabolite concentrations between BCL and BTL groups from the lesion side were all higher in the BCL animals after 1 month and then in the BTL after 4 months. The same regularity was observed for the contralateral side. In small lesions the mean metabolite concentration was higher in SCL for all metabolites in both hemispheres after 1 month. After 4 months all concentrations were higher in SCL only on the contralateral side and NAA+NAAG, Cr+PCr, Glu+Gln on the lesion side. The rest of the metabolites (Ins, Tau, GPC+PCh) were found to have higher mean concentration in STL group.

### Correlation Between Lesion Size Changes and Metabolic Profiles in the Ischemic Rat Brain

**Figure 9:**
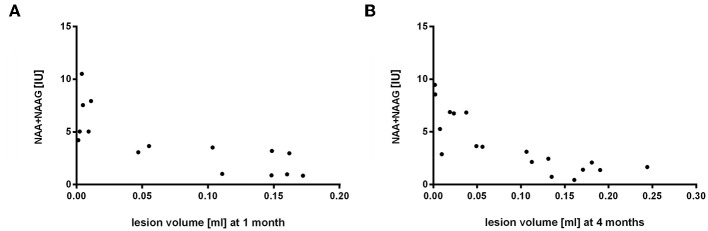
Correlation of volumetry to spectroscopy results from the lesion side 1 and 4 months after MCAO. Lesion volume [ml] and NAA+NAAG concentrations [IU] were compared by Spearman correlation (1 month: *r* = −0.83, *p* = 0.0002; 4 months: *r* = −0.87, *p* = 0.000002).

The aim of this part of the experiment was to determine a possible correlation between volume changes and metabolic profiles in lesioned and contralateral areas over a 4-months period after occlusion and cell injection. These changes at different time points were compared using non-parametric Spearman correlation test. Spectroscopic results from lesion side correlated with volumetric measurements with the highest correlation observed in both time points for NAA+NAAG (1 month: *r* = −0.83, *p* = 0.0002; 4 months: *r* = −0.87, *p* = 0.000002) (Figure 9). Other metabolites on the lesion side significantly correlating with lesion volume were Ins (4 months: *r* = −0.61, *p* = 0.0067), Tau (1 month: *r* = −0.72, *p* = 0.003), GPC+PCh (1 month: *r* = −0.71, *p* = 0.003), Cr+PCr (1 month: *r* = −0.75, *p* = 0.001; 4 months: *r* = −0.76, *p* = 0.0003), and Glu+Gln (1 month: *r* = −0.77, *p* = 0.0007; 4 months: *r* = −0.74, *p* = 0.0005). No correlation was found between spectroscopic results from contralateral side and lesion volume.

## Discussion

A large number of iPSC-differentiated derivatives have already been obtained and successfully used in experimental models of different disorders (34–37), including different stroke models (6, 8, 9). The data in this study may be valuable in elucidating the action mechanisms of neural precursors derived from human iPSCs in stroke models. Using a rat MCAO model, we previously demonstrated that iPSC-NPs (IMR90) successfully alleviate stroke-induced dysfunction. Transplanted neural precursors reversed stroke-induced sensorimotor and motor deficits and also dramatically protected the host substantia nigra from atrophy (16). However, these effects applied only to rats with small lesions, whereas animals with big lesions demonstrated no functional improvement. In this current study, we confirm the promising use of human iPSC-NPs for post-ischemic tissue repair in animals with big lesions. Our histology analysis reveals that transplanted cells survived and migrated toward the lesion area during the 4 month-period after transplantation. They then integrated into the striatal tissue and underwent further differentiation into more mature and tissue-specific neurons. No tumor formation was observed throughout the entire experiment.

Potential recovery and/or other changes were also quantified by volumetric measurements and metabolic changes. The biggest changes in lesion volume means per group were in the small lesion groups. In the SCL group, the volume of ischemic brain lesions decreased throughout the experiment (7 days, 1 month, 4 months), becoming almost invisible on MR images (0.004 ± 0.003 ml) 4 months after MCAO. This effect may be connected with spontaneous recovery of less extensively damaged brain tissue not subjected to invasive transplantation. In STL subjects that did undergo the transplantation procedure, lesion volume also decreased during the first part of the experiment (between 7 days and 1 month: 0.006 ± 0.009−0.002 ± 0.0008) but then increased (between 1 and 4 months: 0.002 ± 0.0008–0.011 ± 0.007). The reason for this difference may be a secondary effect of brain tissue damage arising from cell transplantation and/or inflammation. In the big lesion groups, lesion volume changes were more variable. In BCL animals, lesion sizes increased, with signals becoming more stable between 1 and 4 months (0.125 ± 0.07–0.145 ± 0.09), which may have been caused by a greater range of tissue damage or anoxic depolarization (38). In the BTL group, lesion sizes increased at the beginning but then decreased, with the restoration of the brain cells functions perhaps attributable to cell grafts integrating into the tissue at this time point. Our hypothesis to explain the initial lesion size increase followed by a decrease is that the necrotic processes are slowing down in time and grafted cells need time to incorporate to the lesioned tissue, maybe more time points would standardize these results. All of these results suggest that, in the case of big lesions, spontaneous recovery and compensation of ischemic damage in the brain is limited.

Spectroscopy is a non-invasive method used to precisely determine biochemical changes in damaged tissue. In addition to lesion volume alterations, we also observed changes in metabolite concentrations in our animal model. As with volumetry analysis, results were again more variable in the case of big lesions. The first round of statistical analysis involved obtaining data on metabolite concentrations from the ipsilesional and contralateral hemispheres as a control for themselves. In the BTL group, a significant difference (*p* < 0.01) in Tau, NAA+NAAG, Cr+PCr, Glu+Gln concentrations were observed 4 months after MCAO, with higher values on the contralateral side. According to the literature, taurine has the ability to maintain neuronal calcium homeostasis and prevent neuronal cell death due to necrosis or apoptosis (24). Taurine may also be used in ischemic stroke, during which intracellular free calcium increases. Levels of taurine are understood to increase in patients after stroke (25, 39). An increase in glutamate concentration is considered toxic, as it binds to ionotropic N-methyl-D-aspartate (NMDA) and α-amino-3-hydroxy-5-methyl-4-isoxazolepropionic acid (AMPA) receptors. This in turn promotes a major influx of calcium, which triggers phospholipases and proteases that degrade essential membranes and proteins (40). Tissue necrosis may have been suppressed with the neuroprotective influence from Tau, NAA+NAAG and Cr+PCr. Confirming these findings in our study, we observed a decrease in lesion volume co-occurring with higher metabolite concentrations after 4 months in both hemispheres. Previous studies have identified lower concentrations of the neuronal marker NAA (27) in lesions (41) and the ability of Cr to protect the brain from neurotoxicity (28). All mentioned metabolites had also higher (on the limit of statistical significance) concentrations on the contralateral side 1 month after MCAO. Here additional might be the functional improvement induced by GPC, already mentioned in models of ischemic damage (42).

In the BCL group results which reflect the direction of changes (1 month: Cr+PCr, Glu+Gln; 4 months: Tau, NAA+NAAG, Cr+PCr, Glu+Gln) were found, all with concentrations higher on the contralateral side. In all four groups in our study, metabolite concentrations were lower on the lesion side.

In the second round of statistical analysis, differences occurring over time on both contralateral and lesion sides were measured. In BLT group metabolites concentrations changed—this time on the contralateral side, with significant results in all metabolites. In our study, the contralateral side probably compensated for the decrease in lesion metabolites concentration and may have influenced the decrease in lesion volume, as we observed in BTL and SCL groups. Here we can point out also trend of results observed in the BTL group, with Cr+PCr concentrations higher on the lesion side 4 months after MCAO compared to the initial measurement. This may have influenced the recovery of brain tissue, as lesion volume also decreased during this time. The functions of creatine and phosphocreatine in improving cerebrovascular function and reducing lesion have been previously reported (43).

Based on group comparisons, the biggest differences in metabolites were found 1 month following transplantation. However, no significant results in metabolite concentrations between same lesion size groups was obtained, in the BTL group all metabolite concentrations from both hemispheres were lower than those in the BCL group after 1 month and all higher after 4 months. In the STL group, the same trend was observed on the lesion side for Ins, Tau and GPC+PCh.

The glutamate concentrations are higher on the contralateral side possibly because there is no tissue necrosis in this hemisphere after 1 and 4 months and as there is healthier neural tissue, we can observe higher glutamate levels. If the measurement would be performed in shorter period after the MCAO procedure was carried out, there would be probably more glutamate measured on the lesion side, as the changes in the tissue structure were not so drastic. Our results show that bigger mean differences between hemispheres in glutamate concentrations were observed in big lesions (Table 2B), what could support our hypothesis.

The lower metabolite concentrations in treated animals we observed were probably due to the inherent fluctuation of transplanted cells and the injections, an invasive procedure that can lead to biochemical changes. In all animals, in which lesion volume decreased at the onset of ischemic damage, the protective function of the penumbra could be observed (44). Its possible influence highlights the importance of introducing additional measurements during early stages of lesion development.

Based on our literature search, our data are not in contradiction with published MRS results (45). However, it should be mentioned that published MRS data are usually obtained from MR scanners with higher magnetic field (45), which affect significantly relaxation times, or from different species (46). Therefore, concentration of metabolites can be slightly different.

The findings of this study have to be seen in light of some limitations. The first is the absence of sham operated group of animals and group of animals transplanted with vehicle. Our interpretation of the above results is possibly limited by the small group sizes and the time discrepancy between measurements of MRS (taken 1 and 4 months after MCAO) and MRI (initially taken 14 days after MCAO). Even the smallest changes in time can have a crucial influence on stroke, a factor that may reflect our results. It is necessary to mention that metabolite concentrations were assessed by LCModel without T1, T2 correction factor. TR = 2,500 ms was chosen as a compromise between accuracy and scan time (to minimize passing of animal during scanning). If we look at the relaxation times of metabolites [for example (47) or (48)], we can roughly estimate that real absolute metabolite concentration would be about 20% higher. Higher effect has T1 relaxation time, attenuation of MRS signal due to T2 is not high because T2 relaxation times of metabolites are much higher than used TE (13 ms) (47, 48). Due to the technical reasons, we were not able to quantify the numbers of surviving and differentiated grafted iPSC-NP cells.

## Conclusion

We confirm the beneficial effect of induced pluripotent stem cell-derived neural precursors on post-ischemic tissue repair in animals with small lesions (demonstrated previously) as well as in those with big lesions. We suggest that iPSC-NP transplantation leads to a decrease in lesion volume and helps to regulate metabolite concentrations within the normal range expected in healthy nervous tissue. These results may add to the current store of knowledge on the biochemical processes occurring during early stages of ischemic stroke. Studying the correlation of lesion volume changes with spectroscopic changes over time may improve our understanding of how metabolite concentrations change after occlusion and how they are linked to lesion volume, leading to better predictions of stroke outcomes. This may in turn benefit early diagnosis and elucidate the dynamic pathogenesis of early cerebral ischemia. The decrease in lesion volume we observed in animals with big lesions indicates that iPSC-NP transplantation may indeed be a safe tool for cell transplantation therapy in stroke and a promising tool for restoring neurological function. However, how the differentiation of iPSC-NPs into tissue-specific neurons leads to the long-term restoration of lesioned tissue precisely needs to be researched in more details. As most of the major changes occur at stroke onset, particular attention should be given to the metabolite and morphologic changes that occur during early stages of stroke.

## Data Availability Statement

All datasets generated for this study are available on request.

## Ethics Statement

All animal experiments were performed in accordance with EU Directive 2010/63/EU on the protection of animals used for scientific purposes, and approved by the ethics committees of the Institute of Experimental Medicine, the Czech Academy of Sciences, and the Institute for Clinical and Experimental Medicine.

## Author Contributions

DJ: performance and coordination of experiments, MRI and MRS measurements, spectroscopy data processing, statistical analysis, and manuscript writing. NZ: volumetry and MRI and MRS statistical analysis. KT: animal surgery (MCAO), behavioral testing, and data and statistical analysis. KK: RNA isolation, PCR, and data and statistical analysis. ES: financial support and project management. PJ: experimental design, project management, critical reading, and correction of the manuscript. NR: experimental design, performance and coordination of experiments, iPSC-NP culturing and grafting, immunocytochemical analysis, animal surgery (MCAO), immunohistochemical analysis, confocal imaging, data and statistical analysis, and manuscript writing.

### Conflict of Interest

The authors declare that the research was conducted in the absence of any commercial or financial relationships that could be construed as a potential conflict of interest.

## Supplementary Material

### Methods

#### RNA Extraction and Quantitative PCR Analysis of Gene Expression

Brain tissue was dissected from animals of two different groups: control animals with big lesions that underwent the MCAO procedure only (*n* = 4) and animals with big lesions grafted with iPSC-NPs (*n* = 4) sacrificed 4 months after transplantation. Total RNA was extracted from rat brain tissue (frozen sections) using the High Pure RNA Paraffin Kit (Roche, Penzberg, Germany) according to the manufacturer's instructions. Reverse transcription was performed using the T100™ Thermal Cycler (Bio-Rad, Hercules, CA, USA) and the Transcriptor Universal cDNA Master (Roche, Penzberg, Germany).

Expressions of the rat (Rattus norvegicus) target genes Bdnf, Ngf, Vegf, Fgf8, Dcx, Syp, Ppp1r1b, Drd1, Drd2, Gap43, and Actb were determined by quantitative real-time reverse transcription polymerase chain reaction (qPCR) using StepOnePlus™ (Life Technologies, Carlsbad, CA, USA), the FastStart Universal Probe Master (Roche, Penzberg, Germany) and TaqMan Gene Expression Assays (4331182) [Rn02531967_s1/Bdnf/, Rn01533872_m1/Ngf/, Rn01511601_m1/Vegfa/, Rn00590996_m1/Fgf8/, Rn00670390_m1/Dcx/, Rn01528256_m1/Syp/, Rn01452984_m1/Ppp1r1b/, Rn03062203_s1/Drd1/, Rn00561126_m1/Drd2/, Rn01474579_m1/Gap43/, Rn00667869_m1/Actb/]. The final reaction volume amounted to 10 μL containing 25 ng of extracted RNA. The following thermal profile was used: a single cycle to prevent carryover contamination for 2 min at 50°C and 10 min at 95°C for DNA polymerase activation, followed by 40 cycles of denaturation at 95°C for 15 s and annealing and extension at 60°C for 1 min. The results were analyzed using integrated StepOnePlus^®^ software (Life Technologies, Carlsbad, CA, USA). Each data set was normalized with an appropriate TaqMan endogenous control selected by NormFinder. Actb [Rn00667869_m1] was chosen as the housekeeping gene. Finally, data were recalculated to relative quantities and transformed to a log_2_ scale.

### Results

To evaluate the effect of transplanted iPSC-NPs on endogenous regenerative processes, we examined the gene expression of several neurotrophic factors and the neurospecific genes Bdnf, Ngf, Vegfa, Fgf8, Dcx, Syp, Ppp1r1b, Drd1, Drd2, and Gap43 separately in the right (lesioned) and left hemispheres of control and transplanted animals 4 months after cell grafting using qPCR. This analysis was performed only for animals with big lesions; similar results for animals with small lesions are presented in Polentes et al. (16). We found no significant differences in the expression of listed genes in transplanted animals vs. control animals with big lesions (Supplementary Figure 4).

Supplementary Figure 1 Graft survival and localization in rat ischemic brains with small and big lesions showing immunohistochemical staining with MTC02, a marker of human mitochondria; magnification −1 mm.

Supplementary Figure 2 Immunohistochemical staining of ischemic rat brain tissue with small lesion size 4 months after iPSC-NP transplantation. Grafted human neural precursors were identified by the staining for MTC02 **(A,B)**. They differentiated into NeuN- **(E)**, MAP2- **(F)** and DARPP32- **(D)** positive cells, actively integrated into the lesioned host tissue **(C,G)**.

Supplementary Figure 3 Metabolite concentrations [IU] ± std for healthy animals.

Supplementary Figure 4 Effect of transplanted iPSC-NPs on rat gene expression 4 months after MCAO.
