# Supplementary figures and images for: Metabolic Changes in Focal Brain Ischemia in Rats Treated With Human Induced Pluripotent Stem Cell-Derived Neural Precursors Confirm the Beneficial Effect of Transplanted Cells
Source: Front Neurol. 2019 Oct 22;10:1074. doi: 10.3389/fneur.2019.01074 (PMC6818685; doi:10.3389/fneur.2019.01074)

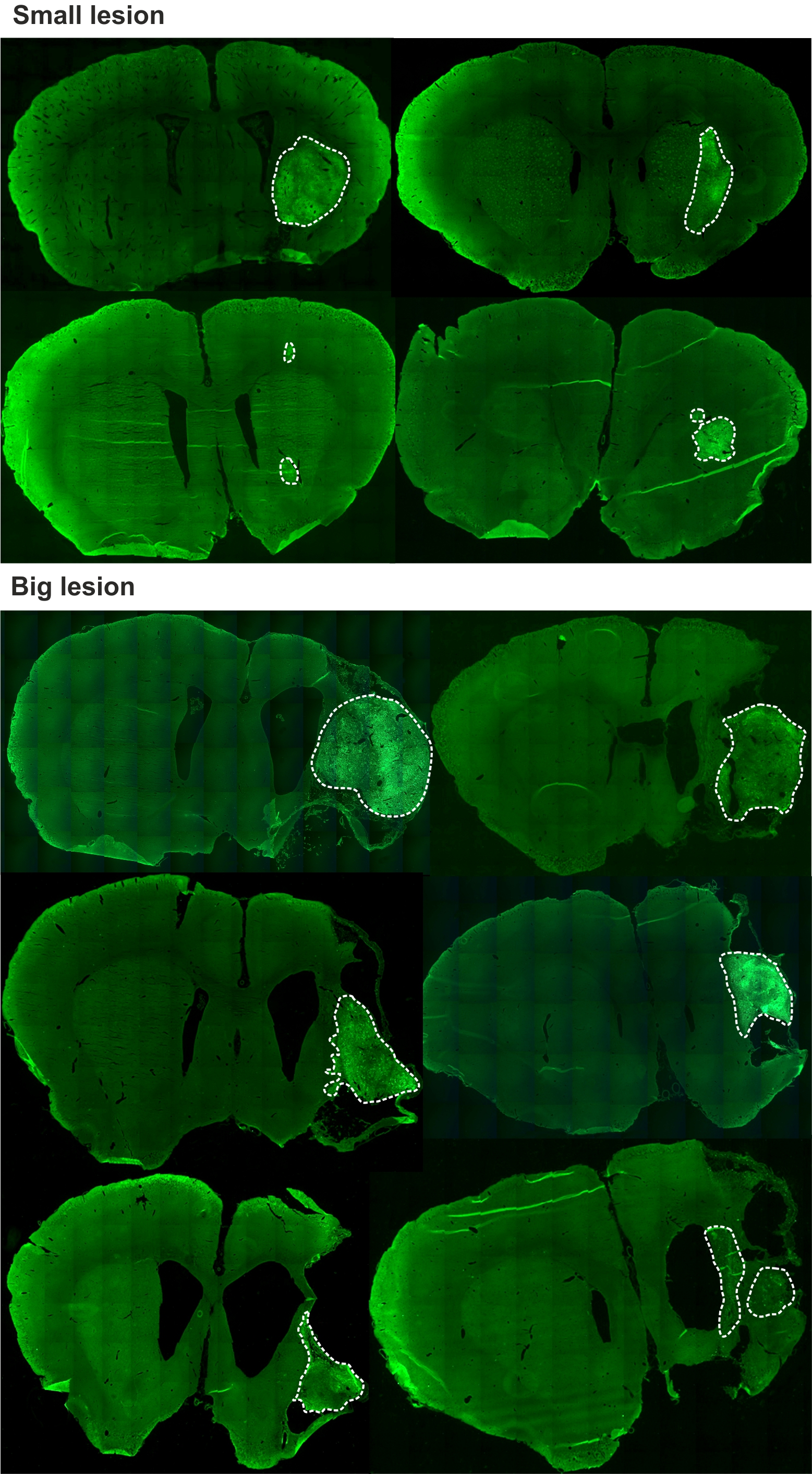

Supplement: Supplementary Figure 1 — Graft survival and localization in rat ischemic brains with small and big lesions showing immunohistochemical staining with MTC02, a marker of human mitochondria; magnification −1 mm. [file Image_1.JPEG]

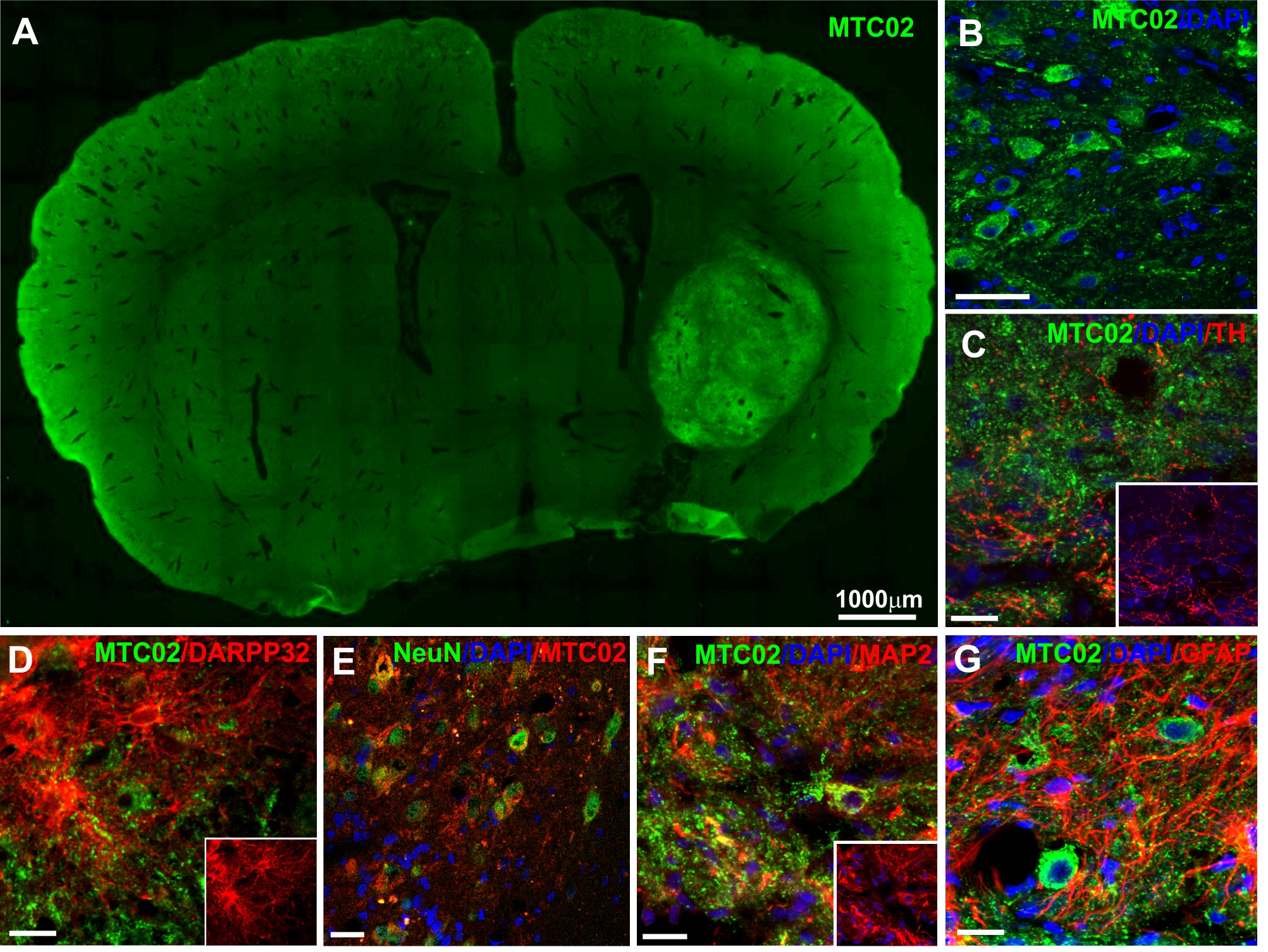

Supplement: Supplementary Figure 2 — Immunohistochemical staining of ischemic rat brain tissue with small lesion size 4 months after iPSC-NP transplantation. Grafted human neural precursors were identified by the staining for MTC02 (A,B). They differentiated into NeuN- (E), MAP2- (F) and DARPP32- (D) positive cells, actively integrated into the lesioned host tissue (C,G). [file Image_2.JPEG]

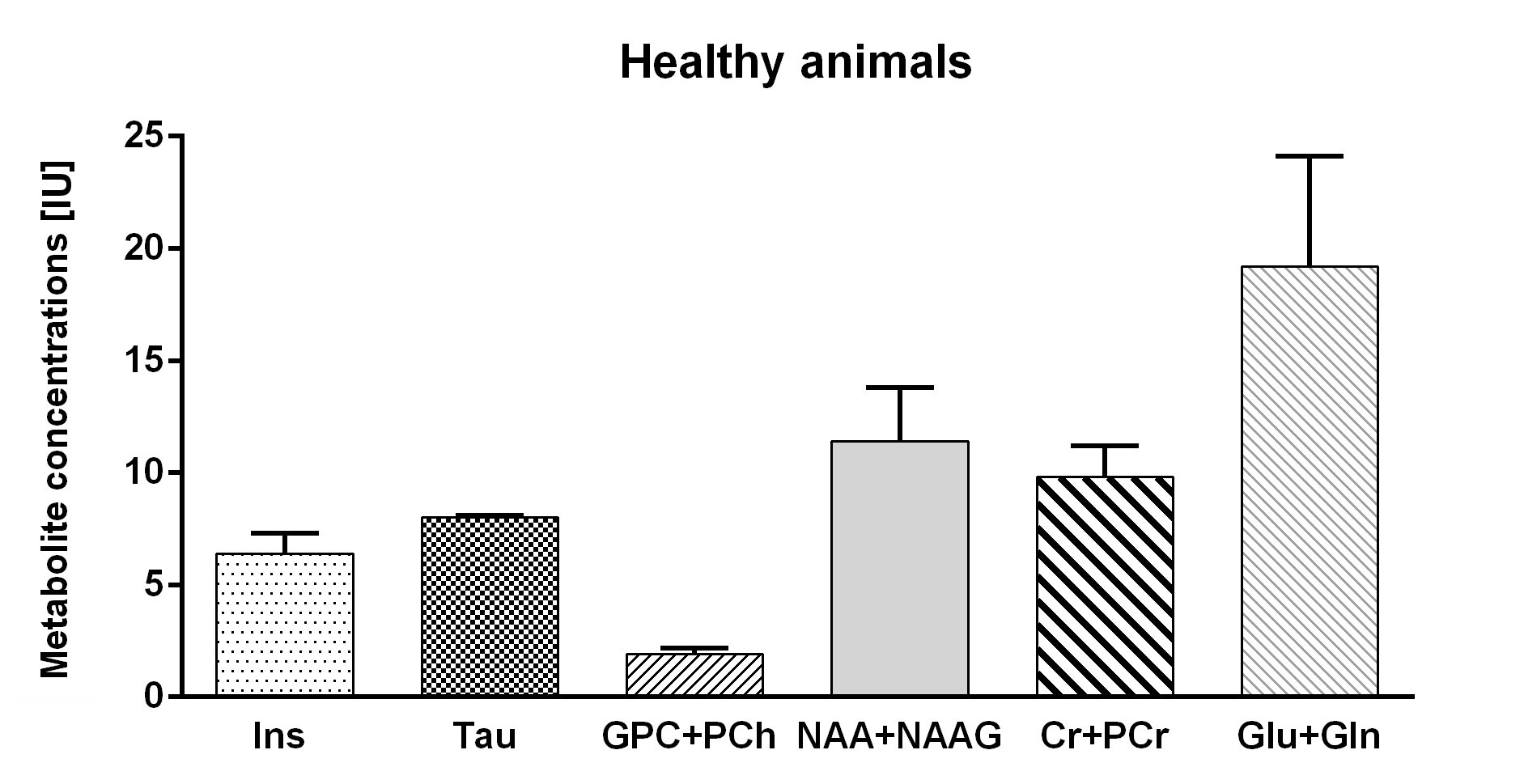

Supplement: Supplementary Figure 3 — Metabolite concentrations [IU] ± std for healthy animals. [file Image_3.JPEG]

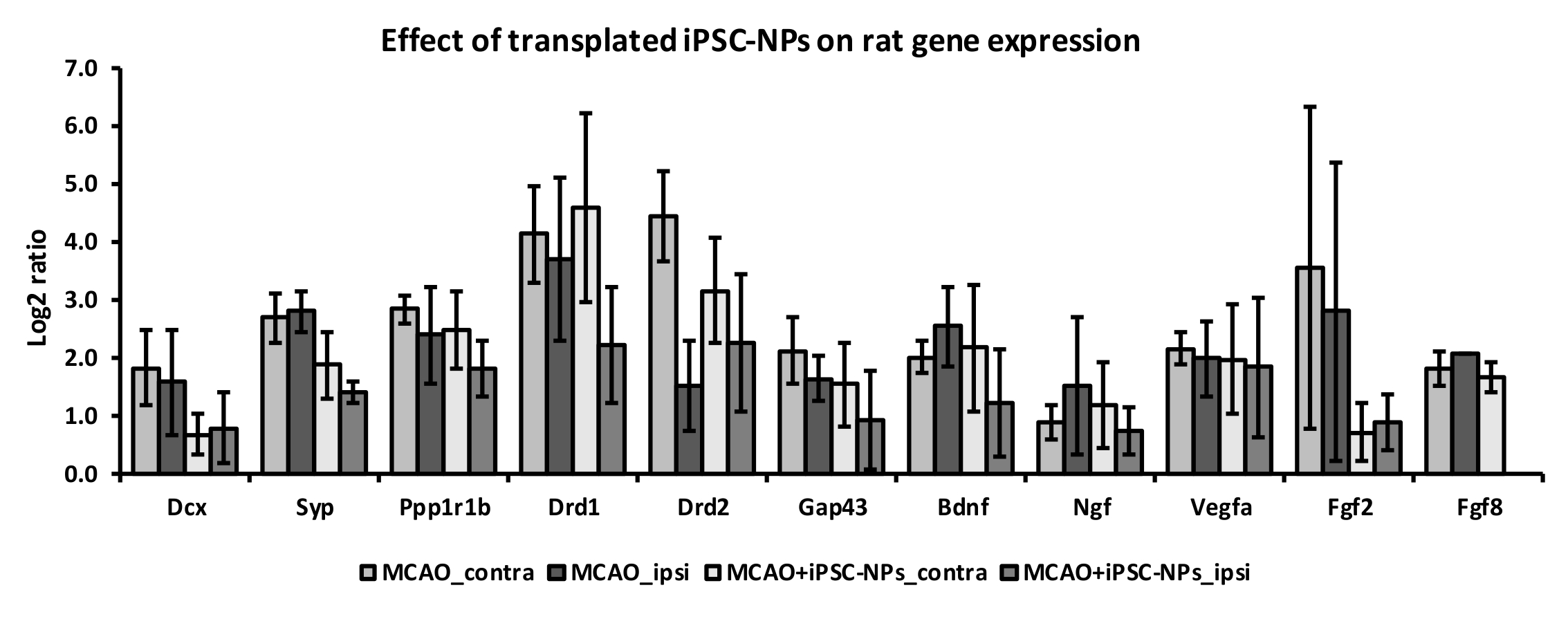

Supplement: Supplementary Figure 4 — Effect of transplanted iPSC-NPs on rat gene expression 4 months after MCAO. [file Image_4.JPEG]
